# RyR2 regulates store-operated Ca^2+^ entry, phospholipase C activity, and electrical excitability in the insulinoma cell line INS-1

**DOI:** 10.1371/journal.pone.0285316

**Published:** 2023-05-04

**Authors:** Kyle E. Harvey, Shiqi Tang, Emily K. LaVigne, Evan P. S. Pratt, Gregory H. Hockerman

**Affiliations:** 1 Department of Medicinal Chemistry and Molecular Pharmacology, Purdue University, West Lafayette, Indiana, United States of America; 2 Purdue Interdisciplinary Life Sciences Program, Purdue University, West Lafayette, Indiana, United States of America; Cinvestav-IPN, MEXICO

## Abstract

The ER Ca^2+^ channel ryanodine receptor 2 (RyR2) is required for maintenance of insulin content and glucose-stimulated insulin secretion, in part, via regulation of the protein IRBIT in the insulinoma cell line INS-1. Here, we examined store-operated and depolarization-dependent Ca^2+^entry using INS-1 cells in which either RyR2 or IRBIT were deleted. Store-operated Ca^2+^ entry (SOCE) stimulated with thapsigargin was reduced in RyR2^KO^ cells compared to controls, but was unchanged in IRBIT^KO^ cells. STIM1 protein levels were not different between the three cell lines. Basal and stimulated (500 μM carbachol) phospholipase C (PLC) activity was also reduced specifically in RyR2^KO^ cells. Insulin secretion stimulated by tolbutamide was reduced in RyR2^KO^ and IRBIT^KO^ cells compared to controls, but was potentiated by an EPAC-selective cAMP analog in all three cell lines. Cellular PIP_2_ levels were increased and cortical f-actin levels were reduced in RyR2^KO^ cells compared to controls. Whole-cell Ca_v_ channel current density was increased in RyR2^KO^ cells compared to controls, and barium current was reduced by acute activation of the lipid phosphatase pseudojanin preferentially in RyR2^KO^ cells over control INS-1 cells. Action potentials stimulated by 18 mM glucose were more frequent in RyR2^KO^ cells compared to controls, and insensitive to the SK channel inhibitor apamin. Taken together, these results suggest that RyR2 plays a critical role in regulating PLC activity and PIP_2_ levels via regulation of SOCE. RyR2 also regulates β-cell electrical activity by controlling Ca_v_ current density and SK channel activation.

## Introduction

Temporal and spatial regulation of intracellular Ca^2+^ concentrations ([Ca^2+^]_in_) is critical for pancreatic β-cell function and survival [1], and aberrant Ca^2+^ handling is associated with type 2 diabetes [2]. Elevation of [Ca^2+^]_in_ in β-cells involves both influx of Ca^2+^ into cells via plasma membrane resident channels [1], and release of Ca^2+^ from the endoplasmic reticulum (ER) [3]. Influx of Ca^2+^ through voltage-gated Ca^2+^ (Ca_v_) channels is an essential step for maximal insulin secretion by pancreatic β-cells [4]. Ca^2+^ influx is amplified by activation of channels that release Ca^2+^ from internal stores through the process of Ca^2+^-induced Ca^2+^ release (CICR) [3, 5]. Voltage-gated Ca^2+^ entry is mediated through Ca_v_1.2, Ca_v_1.3, and Ca_v_2.1 in β-cells, and these ion channels are essential to insulin secretion and beta-cell survival [1, 4, 6]. While CICR is not essential for depolarization-induced insulin secretion, it is thought to amplify the effect [5, 7].

The two major classes of Ca^2+^ release channels residing on the ER membrane of β-cells are ryanodine receptors (RyR) and inositol 1,4,5-trisphosphate receptors (IP_3_R) [8]. RyR2 is expressed in human [9] and mouse β-cells [10], and is the predominant, if not sole, RyR functionally expressed in the rat insulinoma cell line INS-1 [11]. In addition to Ca^2+^ binding, RyR2 is activated by the second messengers cyclic ADP ribose and nicotinic acid adenine dinucleotide phosphate [12–14]. IP_3_R3 is the predominant IP_3_R expressed in human β-cells [8] and rat islets [15], but IP_3_R1 is predominant in mouse islets [16]. Dysregulation of Ca^2+^ homeostasis leads to ER stress and pancreatic β-cell death [17, 18]. ER Ca^2+^ depletion occurs under prolonged exposure to cytokines, fatty acids, and inhibition of sarcoplasmic/endoplasmic reticulum Ca^2+^ ATPase (SERCA) [19]. Store-operated Ca^2+^ entry (SOCE) is a mechanism by which ER Ca^2+^ stores are replenished by capacitative calcium entry, in contrast to depolarization-dependent Ca^2+^ influx [20]. Ca^2+^ release-activated channels (CRAC) mediate SOCE and are activated when ER Ca^2+^ is depleted. Upon a drop in ER Ca^2+^, the Ca^2+^ sensor STIM,1 present on the on the cytosolic face of the ER, translocates to ER-plasma membrane junctions. Upon translocation, STIM1 interact with Orai and TRPC1 allowing for influx of extracellular Ca^2+^ [21, 22]. SOCE is an essential process for refilling intracellular Ca^2+^ stores in non-excitable cells (i.e. lacking voltage-gated Ca^2+^ channels), but is also functionally important in excitable cells [23]. Islets from type 2 diabetics were shown to have reductions in STIM1, and deletion of STIM1 from INS-1 pancreatic β-cells was shown to impair SOCE [24]. Pharmacological inhibition of SOCE inhibits glucose-stimulated insulin secretion, but inhibitors of SOCE have many off-target effects [24–26]. RyRs and IP_3_Rs may play a role in activation of SOCE. In pulmonary artery smooth muscle cells, stimulation of RyR2 can activate SOCE via a mechanism dependent on ER/SR Ca^2+^ depletion and a specific conformation of RyR2 [27]. IP_3_R may also play a role in the activation of SOCE in β-cells. Depletion of ER calcium stores by muscarinic receptor activation was sufficient to activate SOCE in INS-1E cells, and IP_3_R has direct and indirect interactions with various TRPC channels, including TRPC1 [25].

One potential role for SOCE, besides re-filling ER Ca^2+^ stores upon Ca^2+^ release via RyR and IP_3_R, is to provide Ca^2+^ to maintain phospholipase C (PLC) activity. PLCs are Ca^2+^-dependent enzymes that catalyze the hydrolysis of phosphatidyl inositol-4,5-bisphophate (PIP_2_) to inositol 1,4,5-trisphosphate (IP_3_) and diacylglycerol in the basal state, or upon stimulation by G_q_-coupled receptors [28]. SOCE plays a stimulatory role on prolonged PLC activity in response to the muscarinic receptor agonist carbachol in both MIN6 insulinoma cells and primary mouse pancreatic β-cells [29]. In these studies, 2-aminoethyl diphenylborate (2-APB), a small-molecule inhibitor of SOCE, acutely inhibited the late, sustained phase of PLC activity, but inhibition of membrane depolarization with diazoxide or inhibition of L-type VGCC with nifedipine was without effect. Another potential consequence of reduced PLC activity, particularly basal activity, is chronically increased PIP_2_ levels in the plasma membrane. An accumulation of PIP_2_ could have multiple effects on electrical activity in excitable cells since it has direct modulatory effects on ion channel activity [30], including that of Ca_v_ channel in β-cells [31].

We have previously demonstrated that crosstalk between RyR2 and IP_3_R is partially mediated through regulation of expression of IRBIT (IP_3_ Receptor Binding protein released with Inositol 1,4,5 Trisphosphate (a.k.a. AHCYL1)), as deletion of RyR2 from INS-1 cells leads to reduced levels of the protein IRBIT and subsequent dysregulation of IP_3_R [11]. Deletion of RyR2 also leads to marked reduction in insulin transcript, content, and glucose-stimulated secretion [11]. In the present study, we investigated the functional consequences of deletion of RyR2 and IRBIT on Ca^2+^ signaling via examination of SOCE, PLC activity, and electrical activity in control INS-1 cells and INS-1 cells in which RyR2 or IRBIT have been deleted using CRISPR/Cas9 gene editing. Our results suggest that RyR2 plays a crucial role in regulation of SOCE and PLC activity independent of IRBIT, as well as in the regulation of electrical activity in β-cells.

## Materials and methods

### Chemicals and reagents

Fura-2 AM was from Molecular Devices (San Jose, CA). Xestospongin C and rapamycin were from Cayman Chemical (Ann Arbor, MI). Bethanechol was from Alfa Aesar (Haverhill, MA). Apamin was from Tocris Bioscience (Bristol, UK). Stim1 antibody and mouse IgG-κ Fc binding protein conjugated to CFL 488 were from Santa Cruz Biotechnology (Dallas, TX). Secondary antibody (goat anti-mouse IgG conjugated to horseradish peroxidase) was from BioRad (Hercules, CA). Antibodies to phosphatidylinositol 4,5 bisphosphate (PIP_2_) were from Echelon Biosciences (Salt Lake City, UT). All other reagents, unless otherwise indicated, were from Sigma-Aldrich (St. Louis, MO). Phalloidin conjugates were from Biotium (Fremont, CA). Plasmids encoding Pseudojanin (PJ) (Addgene plasmid # 37999), LYN11-FRB-CFP (Addgene plasmid # 38003), and PJ-DEAD (PJ-D) (Addgene plasmid # 38002) were gifts from Robin Irvine. GFP-C1-PLCdelta-PH was a gift of Tobias Meyer (Addgene plasmid # 21179).

### Cell lines

INS-1 cells [32] (gifted by Dr. Ming Li, Tulane University) were cultured in RPMI-1640 medium (Sigma-Aldrich, St. Louis, MO) supplemented with 10% fetal bovine serum (Qualified, Gibco), 11 mg/mL sodium pyruvate, 10 mM HEPES, 100 U/mL penicillin, 100 μg/mL streptomycin, and 50 μM mercaptoethanol at 37°C, 5% CO_2_. Construction and characterization of the RyR2^KO^ and IRBIT^KO^ cell lines were described previously [11].

### Single-cell intracellular Ca^2+^ assays

INS-1, RyR2^KO^, and IRBIT^KO^ cells were plated in a poly-D-lysine coated 4-chambered 35 mm glass bottom tissue culture dish (Cellvis, Mountain View, CA). Cells were incubated overnight in RPMI-1640 medium at 37°C, 5% CO_2_. Cells were washed twice with PBS and loaded with 3 μM Fura-2 AM (Thermo Fisher, Waltham, MA) diluted in a modified Krebs-Ringer buffer solution [KRBH: 134 mM NaCl, 3.5 mM KCl, 1.2 mM KH_2_PO_4_, 0.5 mM MgSO_4_, 1.5 mM CaCl_2_, 5 mM NaHCO_3_, 10 mM HEPES (pH 7.4)] supplemented with 0.05% fatty acid free BSA at room temperature for 1 hour. The KRBH-containing Fura-2 AM was removed, cells were washed twice with KRBH, then equilibrated in KRBH alone or KRBH containing a 2X concentration of indicated inhibitors for 30 minutes at room temperature. The 4-chambered 35 mm dish was mounted on a chamber attached to the stage of an Olympus IX50 inverted microscope equipped with a PlanApo 20X objective lens (0.95 na). Cells were stimulated with the indicated stimulus at a 2X concentration. Cells were alternatively excited at 340/11 nm and 380/20 nm wavelengths using a bandpass filter shutter (Sutter Instruments, Novato, CA) and changes in intracellular Ca^2+^ were measured by recording the ratio of fluorescence intensities at 508/20 nm in time lapse with a time interval of 0.6 seconds using a Clara CCD camera (Andor Technology, Belfast, Ireland). Background subtraction from the raw 340/11 nm and 380/20 nm wavelengths was performed, then isolated single cells were selected and traced as regions of interest (ROIs) and the 340/11 nm/380/20 nm ratios were measured for each ROI using MetaMorph image analysis software (Molecular Devices, San Jose, CA). Single-cell Ca^2+^ traces were normalized to their respective baseline intracellular Ca^2+^ level obtained by averaging the 340/11 nm/380/20 nm ratios during the first minute of each experiment in the absence of stimulus. Ca^2+^ transients are plotted as normalized 340/11 nm/380/20 nm ratios against time.

### Intracellular Ca^2+^ measurements in 96-well plates

INS-1, RyR2^KO^, and IRBIT^KO^ cells were plated at 70–90% confluency in black-walled 96-well tissue culture plates (Corning, Corning, NY) in RPMI-1640 medium and incubated overnight at 37°C, 5% CO_2_. Cells were washed twice with PBS and incubated with 100 μL 5 μM Fura-2 AM diluted in KRBH for 1 hour at room temperature. The KRBH containing Fura-2 AM was removed, cells were washed twice with KRBH, then equilibrated for 30 minutes at room temperature. To measure SOCE, baseline fluorescence was measured for 1 minute. Thapsigargin was then injected to a final concentration of 1 μM to deplete ER Ca^2+^ stores and fluorescence was measured for 20 minutes. 2-APB was co-injected with thapsigargin in some experiments to a final concentration of 100 μM to block SOCE. Finally, a final concentration of 2.5 mM CaCl_2_ was injected, and fluorescence measured for 10 minutes. To measure fluorescence, cells were alternatively excited at 340/11 nm and 380/20 nm (center/bandpass) and changes in intracellular Ca^2+^ concentrations were measured by recording the ratio of fluorescence intensities at 508/20 nm (15 second time interval) using a Synergy 4 multimode microplate reader (BioTek, Winooski, VT). Traces were normalized to their respective baseline intracellular Ca^2+^ level obtained by averaging the 340/11 nm/380/20 nm ratios of each experiment in the absence of stimulus. Ca^2+^ transients are plotted as normalized 340/11 nm/380/20 nm ratios against time.

### IP_1_ HTRF assays

INS-1, RyR2^KO^, and IRBIT^KO^ cells were plated at approximately 200,000 cells/well in a white opaque 96-well tissue culture plate (Corning, Corning, NY) and incubated overnight in low glucose RPMI-1640 medium at 37°C, 5% CO_2_. Cells were washed twice with PBS and incubated in a pre-stimulation buffer [10 mM HEPES, 1 mM CaCl_2_, 0.5 mM MgCl_2_, 4.2 mM KCl, 146 mM NaCl (pH 7.4)] for 1 hour at 37°C, 5% CO_2_. The pre-stimulation buffer was decanted, and stimulants and/or inhibitors at the indicated concentrations were added to the cells in the same pre-stimulation buffer supplemented with 50 mM LiCl to inhibit inositol monophosphate degradation and incubated for 1 hour at 37°C, 5% CO_2_. Accumulation of IP_1_ was measured using the IP-One Gq Homogenous Time-Resolved Fluorescence (HTRF) kit from Perkin Elmer (Waltham, MA), per the manufacturer’s instructions. The IP_1_ concentration of each sample was interpolated by comparison to a standard curve of known IP_1_ concentrations.

### Insulin secretion assays

INS-1, RyR2^KO^, and IRBIT^KO^ cells were plated at 70–90% confluency in 96-well plates (Corning) in RPMI-1640 media and incubated overnight at 37°C, 5% CO_2_. 16–24 h prior to assay, cells were incubated in serum-free, low glucose RPMI-1640 media supplemented with 0.1% fatty acid-free BSA overnight at 37°C, 5% CO_2_. Cells were washed once with PBS and pre-incubated with 100 μL fatty acid-free KRBH alone or containing the working concentration of ESCA for 30 min at 37°C, 5% CO_2_. After 30 min, KRBH was removed and replaced with either 100 μL KRBH or KRBH containing the indicated concentrations of stimulants, and cells were stimulated for 30 min at 37°C, 5% CO_2_. Supernatants were collected and stored at -20°C until assayed. Cells were lysed in 50 μL ice-cold modified RIPA lysis buffer (100 mM NaCl, 25 mM Tris pH 8, 1% Triton X-100, 0.5% sodium deoxycholate, 0.1% SDS, 5 mM MgCl_2_, 1 mM CaCl_2_) supplemented with 10 μg/mL DNAse I and protease inhibitors (1 mM 4-(2-aminoethyl) benzenesulfonyl fluoride hydrochloride, 800 nM aprotinin, 50 μM bestatin, 15 μM E-64, 20 μM leupeptin, and 10 μM pepstatin A). Protein content of lysates was measured using the Pierce BCA Protein Assay Kit (Thermo Fisher) per the manufacturer’s instructions. Insulin measurements were performed with Insulin High-Range assay kits (Perkin Elmer).

### Total internal reflection fluorescence microscopy (TIRFm)

INS-1 cells were transfected with 500 ng GFP-C1-PLCdelta-PH [33] in 12-well plates using LipoJet (Signagen, Frederick, MD) per the manufacturer’s protocol. 24 for hours after transfections, cells were split into poly-D-lysine coated 4-chambered 35 mm glass bottom dishes. Cells were imaged the following morning using a Nikon Ti2-E Inverted Microscope equipped with a TIRF illuminator and a Perfect Focus System (PFS) using a 100x Plan Apo Lambda oil objective (NA 1.4) (Nikon, Tokyo, Japan). Cells were preincubated for 30 min in KRBH containing 2.5 mM glucose at 37°C and 5% CO_2_. After 30 min, cells were imaged at room temperature. Images were acquired at a 1 second interval, 200 msec exposure, on a ORCA-FLASH4.0 digital CMOS camera (Hamamatsu, Hamamatsu, Japan). A 1-minute baseline was recorded prior to addition of 2x concentration of carbachol +/- 2-APB, 100 μM final concentration for both compounds.

### Ca_v_ channel current density measurements

INS-1, RyR2^KO^, and IRBIT^KO^ cells were plated at low confluency in 35 mm tissue culture dishes (Corning) and incubated overnight in RPMI-1640 medium, at 37°C, 5% CO_2_. Whole-cell patch clamp recordings were performed using an Axopatch 200B amplifier (Molecular Devices, San Jose, CA). Data were sampled at 10 kHz and filtered at 1 kHz (six-pole Bessel filter, -3dB). Patch pipettes were pulled from borosilicate glass capillaries (VWR, West Chester, PA), with a P-87 micropipette puller (Sutter instruments, Novato, CA). Pipettes were polished with an MF-830 microforge (Narishige, Tokyo, Japan) to an inside diameter of 3–5 μm. The extracellular solution contained (mM): 140 NaCl, 20 CsCl_2_, 10 BaCl_2_ (or CaCl_2_ as indicated), 10 dextrose, 10 sucrose, 1 MgCl_2_, 10 HEPES, pH 7.4, ~350 mOsm. The intracellular solution contained: 180 N-methyl-D-glucamine (NMDG), 12 phosphocreatine, 5 BAPTA, 4 MgCl_2_, 2 Na_2_ATP, 0.5 Na_3_GTP, 0.1 leupeptin, 40 HEPES, pH 7.3, ~320 mOsm. Current traces were elicited with 100 msec steps to voltages ranging from -70 mV to +50 mV in 10 mV increments every two seconds, from a holding potential of -80 mV, with on-line P/-4 leak subtraction, using pClamp 10.7–11.2 software (Molecular Devices). Peak current (in picoAmperes (pA)) for each cell was divided by whole-cell capacitance (in picoFarads (pF)) to calculate current density (pA/pF). V_1/2_ activation values were determined by plotting normalized tail-current amplitudes versus the corresponding 100-millisecond depolarizing voltage steps from -70 to +50 mV, in 10 mV-increments, from a holding potential of -80 mV. The data were fit to the equation, I = 1/(1 + exp((V_1/2_—V)/k)), where k is a slope factor. For experiments measuring the fraction of Ba^2+^ current blocked by nifedipine in INS-1, RyR2^KO^, and IRBIT^KO^ cells, 5 μM nifedipine was applied to cells under voltage-clamp using an RSC 160 perfusion system (Biologic, Grenoble, France) while stepping from a holding potential of -80 mV to +10 mV for 100 ms, every 20 seconds. For experiments using rapamycin activation of Pseudojanin [34], INS-1, and RyR2^KO^ cells were transfected with Lyn 11-FRP-CFP and either mRFP-FKBP-Pseudojanin active (PJ) or mRFP-FKBP-Pseudojanin phosphatase dead (PJ-D) using Lipojet transfection reagent (SignaGen, Frederick, MD). Rapamycin (1 μM) inhibition of current in transfected cells (identified by epifluorescence) was performed as described above for nifedipine block of current.

### Perforated patch current clamp recordings

Electrophysiological measurements of action potential frequencies were performed as described previously [5].

### Data analysis

Ca^2+^ imaging data, IP_1_ HTRF assay data, and insulin HTRF data were analyzed with Prism (9.3) software (GraphPad, San Diego, CA). Electrophysiological data were analyzed using Clampfit (10.7) software (Molecular Devices) and SigmaPlot (11.0) software (Systat Software, Palo Alto, CA). For statistical analysis, P < 0.05 was considered significant.

## Results

### RyR2 contributes to a rapid rise in [Ca^2^]_in_ upon membrane depolarization and suppresses IP_3_ receptor activation

**Fig 1:**
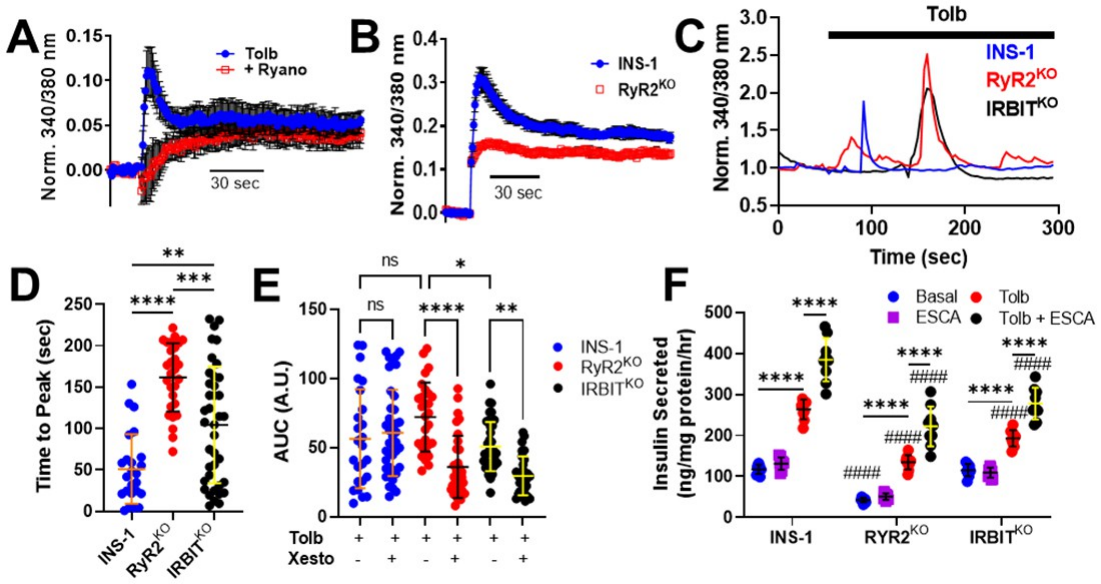
Deletion of RYR2 reshapes the [Ca^2+^]_in_ transient and impairs insulin secretion in response to tolbutamide. **A)** Tolbutamide (200 μM) stimulates a biphasic rise in [Ca^2+^]_in_ in INS-1 cells. Preincubation with ryanodine (100 μM) selectively inhibits the initial peak in the [Ca^2+^]_in_ response. **B)** RyR2^KO^ cells stimulated with tolbutamide show selective loss of the rapid peak of [Ca^2+^]_in_ compared to control INS-1 cells. Experiments in A and B were performed in a 96-well format, over two minutes. Tolbutamide (200 μM) was injected after a 15 sec baseline was recorded. Each point is the mean of triplicate values shown ± SE. **C)** Example traces from single cell [Ca^2+^]_in_ imaging of a control INS-1 cell, an RyR2^KO^ cell, and an IRBIT^KO^ cell over 5 minutes. Tolbutamide was applied at 60 seconds. **D)** Time to greatest peak analysis of INS-1, RyR2^KO^, and IRBIT^KO^ cells. The time to peak was longer for both RyR2^KO^ and IRBIT^KO^ cells compared to INS-1 cell (**P < 0.01; ****P < 0.0001 One-way ANOVA with Tukey’s post-hoc test). The time to peak was longer in RyR2^KO^ compared to IRBIT^KO^ cells (***P < 0.001) One-way ANOVA with Tukey’s multiple comparisons test. INS-1n = 24; RyR2^KO^: n = 28; IRBIT^KO^: n = 39. **E)** Area under the curve analysis (AUC) of the [Ca^2+^]_in_ response to tolbutamide. The AUC was significantly reduced in IRBIT^KO^ cells compared to RyR2^KO^ cells (*, *P* < 0.05), and 1 μM xesto reduced AUC in RyR2^KO^ and IRBIT^KO^ cells only (****, *P* < 00001; **, *P* < 0.01) One-way ANOVA with Tukey’s multiple comparisons test. INS-1: n = 25; INS-1 + xesto: n = 40; RyR2^KO^: n = 28; RyR2^KO^ + xesto: n = 32; IRBIT^KO^: n = 37; IRBIT^KO^ + xesto: n = 30. **F)** Deletion of RyR2 or IRBIT reduces tolbutamide-stimulated insulin secretion compared to INS-1 cells, and reduces insulin secretion stimulated by tolbutamide + ESCA (^####^*P* < 0.0001). Basal insulin secretion is reduced in RyR2^KO^ cells compared to INS-1 cells (^####^, *P* < 0.0001). In all cases, tolbutamide stimulated an increase in insulin secretion over basal (2.5 mM glucose; ****, *P* < 0.0001) and tolbutamide + ESCA stimulated an increase in insulin secretion over tolbutamide alone (****, *P* < 0.0001). n = 9 for all conditions. Two-way ANOVA with Tukey’s multiple comparisons test.

Depolarizing stimuli have been shown to engage CICR in pancreatic β- cells, yet it is not clear whether release of Ca^2+^ from the ER is being mediated by RyR2 or IP_3_R [5]. Membrane depolarization of INS-1 cells with the K_ATP_ channel blocker tolbutamide (200 μM) results in a rapid peak followed by a sustained plateau in [Ca^2+^]_in_ as measured using Fura2-AM in a population of cells with a fluorescence-detecting plate reader, over two minutes (Fig 1A). Pretreatment of INS-1 cells with 100μM ryanodine preferentially inhibited the fast peak of [Ca^2+^]_in_ stimulated by tolbutamide, largely leaving the sustained plateau in [Ca^2+^]_in_ intact (Fig 1A). Deletion of RyR2 from INS-1 cells using CRISPR/Cas9 gene editing abolished the fast peak in [Ca^2+^]_in_ under the same stimulus (Fig 1B). Measurement of changes in [Ca^2+^]_in_ upon stimulation with tolbutamide in single cells over 5 minutes revealed a delay in the time to peak [Ca^2+^]_in_ in RyR2^KO^ cells compared to control INS-1 (Fig 1D). Since we’d previously found that RyR2 deletion results in down-regulation of the protein IRBIT [11], we examined the [Ca^2+^]_in_ response to tolbutamide in INS-1 cells in which IRBIT had been deleted using CRISPR/Cas9 gene editing (IRBIT^KO^ cells). We found that the time to peak [Ca^2+^]_in_ after stimulation with tolbutamide in IRBIT^KO^ cells was longer than in control INS-1 cells, but significantly shorter than in RyR2^KO^ cells. Area under the curve analysis of the tolbutamide-stimulated Ca^2+^ transients showed that deletion of RyR2 didn’t significantly alter the tolbutamide-stimulated Ca^2+^ integral, but deletion of IRBIT reduced the Ca^2+^ integral compared to RyR2^KO^ cells, but not control INS-1 cells. The pretreatment of cells with the IP_3_R blocker xestospongin c (1 μM) reduced the tolbutamide stimulated Ca^2+^ integral in RyR2^KO^ and IRBIT^KO^ cells, but not in control cells (Fig 1E). Tolbutamide is a member of the sulfonylurea class of antidiabetic drugs that is used clinically to stimulate insulin secretion, so we examined tolbutamide-stimulated insulin secretion in control INS-1, RyR2^KO^, and IRBITK^KO^ cells. Basal insulin secretion (2.5 mM glucose) was reduced in the RyR2^KO^ cells compared to control INS-1 cells, and insulin secretion stimulated by 200 μM tolbutamide was significantly reduced in both RyR2^KO^ and IRBIT^KO^ cells compared to control INS-1 cells (Fig 1F). Activation of the cAMP effector protein EPAC2 is thought to potentiate secretion of insulin partially through its ability to mobilize intracellular Ca^2+^ [35]. Therefore, we examined the ability of the cell-permeable EPAC selective cAMP analog CPT-2’-O-Me-cAMP-AM (ESCA; 5 μM) to potentiate tolbutamide-stimulated secretion. We found that ESCA significantly potentiated tolbutamide-stimulated insulin secretion in all three cell lines, but that the magnitude of insulin secretion stimulated by tolbutamide + ESCA was lower in RyR2^KO^ and IRBIT^KO^ cells compared to control INS-1 cells (Fig 1F). Thus, deletion of RyR2 or IRBIT delays the peak of [Ca^2+^]_in_ stimulated by tolbutamide, and permits activation of IP_3_ receptors upon membrane depolarization in INS-1 cells. Further, deletion of RyR2 or IRBIT reduces insulin secretion stimulated by tolbutamide alone or by tolbutamide + ESCA, while deletion of RyR2 sharply reduces basal insulin secretion.

### RyR2 regulates PLC activity in INS-1 cells

**Fig 2:**
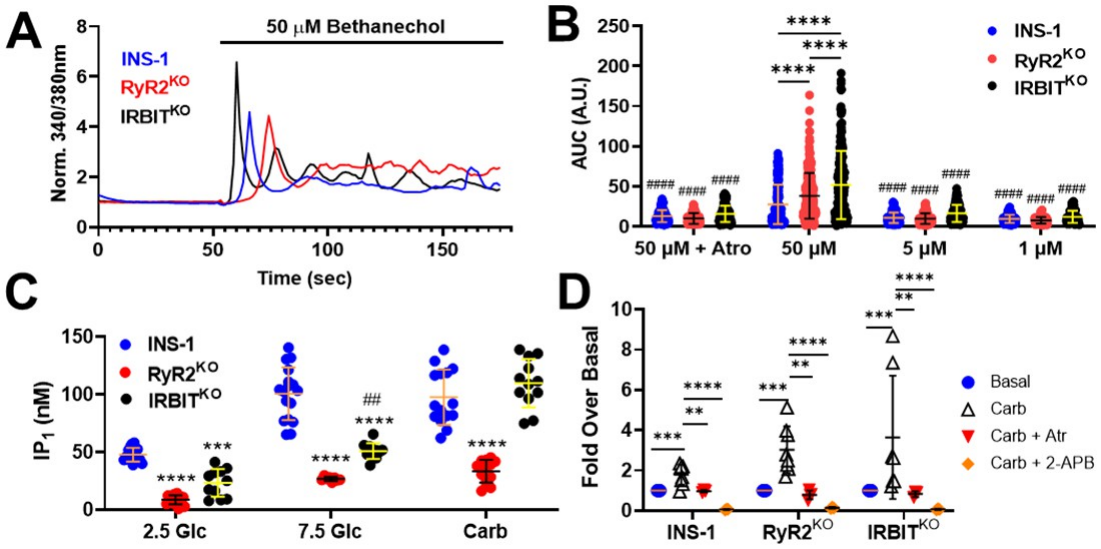
Deletion of RYR2 and IRBIT reduces stimulated and basal PLC activity. **A)** Example traces from single-cell [Ca^2+^]_in_ imaging of a control INS-1 cell, an RyR2^KO^ cell, and an IRBIT^KO^ cell over 3 minutes. Bethanechol was applied at 55–60 seconds. **B)** Quantification of the increase in [Ca^2+^]_in_ stimulated by the muscarinic agonist bethanechol (AUC). 50 μM bethanechol stimulates a greater increase in [Ca^2+^]_in_ in IRBIT^KO^ and RyR2^KO^ cells than in INS-1cells (****, *P* < 0.0001), and a greater increase in IRBIT^KO^ cells than in RyR2^KO^ cells (****, *P* < 0.0001). Stimulation of [Ca^2+^]_in_ by 1 μM or 5 μM bethanechol is not different between the three cell lines, but in each case, is different from that stimulated by 50 μM bethanechol (^####^, *P* < 0.0001). Atropine (100 μM) significantly reduced the [Ca^2+^]_in_ response to 50 μM bethanechol in all cell lines (^####^, *P* < 0.0001) INS-1: 50 μM + Atro n = 175; 50 μM n = 143; 5 μM n = 153, 1 μM n = 132. Ry2R^KO^: 50 μM + Atro n = 164; 50 μM n = 293; 5 μM n = 159, 1 μM n = 111. IRBIT^KO^: 50 μM + Atro n = 106; 50 μM n = 195; 5 μM n = 126, 1 μM n = 102. Outlier analysis was performed using the ROUT method, with Q = 1%. Outliers were removed before statistical analysis. **C)** IP_1_ assay of basal and stimulated PLC activity. Basal (2.5 mM glucose) and 7.5 mM glucose-stimulated PLC activity was reduced in RyR2^KO^ and IRBIT^KO^ cells compared to INS-1 cells (***, *P* < 0.001; ****, *P* < 0.0001). Glucose-stimulated IP_1_ accumulation was greater in IRBIT^KO^ cells than in RyR2^KO^ cells (^##^, *P* < 0;01). In contrast, carbachol (500 μM) stimulated IP_1_ accumulation was significantly reduced in RyR2^KO^ cells compared to both control cells and IRBIT^KO^ cells (****, *P* < 0.0001). Data are shown as mean ±SD. INS-1: 2.5 Glc n = 16, 7.5 Glc n = 17, Carb n = 15; RyR2^KO^: 2.5 Glc n = 13, 7.5 Glc n = 8, Carb n = 14; IRBIT^KO^: 2.5 Glc n = 11, 7.5 Glc n = 11, Carb n = 12. Data for INS-1 cells and RyR2^KO^ cells were previously published in [11], and are shown for comparison with data from IRBIT^KO^ cells. **D)** IP_1_ accumulation stimulated by carbachol in INS-1, RyR2^KO^, and IRBIT^KO^ cells is inhibited by 100 μM atropine and 100 μM 2-APB (*, *P* < 0.05; **, *P* < 0.01; ***, *P* < 0.001; ****, *P* < 0.0001). Data are shown as mean ± SD. Two-way ANOVA with Tukey’s multiple comparisons test. INS-1 cells: basal; n = 8; Carb; n = 8; Carb + Atr; n = 3, Carb + 2-ABP; n = 3. RyR2^KO^ cells: basal, n = 8; Carb, n = 8; Carb + Atr, n = 4; Carb + 2-APB, n = 3. IRBIT^KO^ cells: basal, n = 8; Carb, n = 7; Carb + Atr, n = 3, Carb + 2-APB, n = 3.

Parasympathetic innervation of the pancreas is thought to enhance insulin secretion after meals through activation of muscarinic receptors present on β-cells [36]. Muscarinic receptors M3 and M5 are found in β-cells, and are coupled to Gq signaling, upstream of PLC activation [37]. Therefore, we examined the ability of the muscarinic agonist bethanechol to elevate [Ca^2+^]_in_ in RyR2^KO^, IRBIT^KO^, and control INS-1 cells (Fig 2A). Bethanechol was used in these experiments because it is selective for muscarinic receptors [38], while carbachol also activates nicotinic receptors [39], which could contribute to a Ca^2+^ transient. We compared three concentrations of bethanechol, 1, 5, and 50 μM, to determine if the enhanced activation of IP_3_R observed during tolbutamide stimulation in RyR2^KO^ and IRBIT^KO^ cells enhances bethanechol stimulation of [Ca^2+^]_in_ increases. The Ca^2+^ integrals stimulated by 1 μM or 5 μM bethanechol didn’t differ among the three cell lines, and weren’t different from those observed in the presence of 50 μM bethanechol + 100 μM atropine (Fig 2B). In contrast, 50 μM bethanechol, in the absence of atropine, stimulated a Ca^2+^ transient in all three cell lines that was significantly greater than that stimulated by 1 or 5 μM bethanechol, and the Ca^2+^ integral was greater in both RyR2^KO^ and IRBIT^KO^ cells than in control INS-1 cells (Fig 2B). In addition, the Ca^2+^ integral stimulated by 50 μM bethanechol in IRBIT^KO^ cells was greater than that stimulated in RyR2^KO^ cells (Fig 2B). Thus, deletion of IRBIT or RyR2 enhances the stimulation of [Ca^2+^]_in_ by 50 μM bethanechol in INS-1 cells, but deletion of IRBIT leads to a greater [Ca^2+^]_in_ response to 50 μM bethanechol than deletion of RyR2.

We next examined the ability of 7.5 mM glucose and the muscarinic agonist carbachol (500 μM) to stimulate phospholipase C activity in INS-1, RyR2^KO^, and IRBIT^KO^ cells. For these experiments, we used a homogeneous time-resolved FRET assay for inositol-1-phosphate (IP_1_). In the presence of lithium chloride, IP_1_ is a stable metabolite of IP_3_ and can serve as a surrogate marker for PLC activation and IP_3_ generation [40]. IP_1_ accumulation in 2.5 mM (basal) and 7.5 mM glucose were reduced in both RyR2^KO^ and IRBIT^KO^ cells compared to control INS-1 cells, but IP_1_ accumulation was significantly greater in IRBIT^KO^ cells than in RyR2^KO^ cells in the presence of 7.5 mM glucose (Fig 2C). In contrast, IP_1_ accumulation stimulated by carbachol was only reduced in RyR2^KO^ cells compared to control INS-1 cells (Fig 2C). To ensure that the PLC activity stimulated by carbachol was, in fact, mediated by muscarinic receptor activation, we performed a set of experiments that included inhibition of the response with 100 μM atropine. We found that atropine reduced the accumulation of IP_1_ stimulated by carbachol to a level not different from basal levels (2.5 mM glucose) in all three cell lines (Fig 2D). Store-operated calcium entry (SOCE) is proposed to provide Ca^2+^ to support PLC activity [29]. Accordingly, the application of the SOCE inhibitor 2-APB (100 μM) inhibited carbachol-stimulated IP_1_ accumulation below basal levels in all three cell lines (Fig 2D).

**Fig 3:**
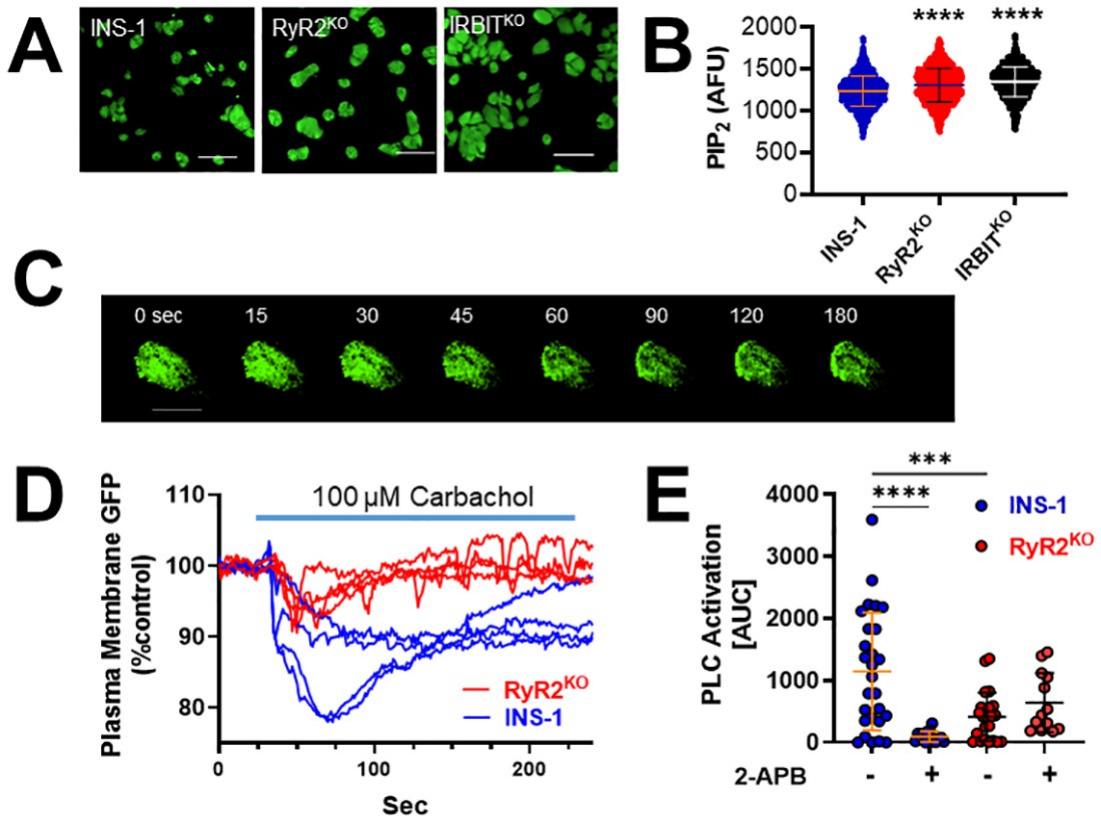
Modulation of PIP_2_ levels by PLC activity in INS-1 and RyR2^KO^ cells. **A)** Micrographs of fixed INS-1, RyR2^KO^, and IRBIT^KO^ cells stained with antibodies to PIP_2_ and IgG-k binding protein conjugated to CFL 488. Scale bars = 50 μm. **B)** Quantification of fluorescence intensity of PIP_2_ immunostaining in control INS-1 cells, RyR2^KO^ cells, and IRBIT^KO^ cells. PIP_2_ staining was greater in RyR2^KO^ cells and IRBIT^KO^ cells compared to control INS-1 cells (****, *P* < 0.0001). INS-1 cells: n = 1294; RyR2^KO^ cells: n = 1377; IRBIT^KO^ cells n = 1650. Data are shown as mean ±SD. One-way ANOVA with Dunnett’s multiple comparisons test. Data for control INS-1 cells and RyR2^KO^ cells were previously published in [11] and are shown for comparison with data from IRBIT^KO^ cells. **C)** Time lapsed TIRFm images of GFP-C1-PLCdelta-PH localization to the plasma membrane upon stimulation with 100 μM carbachol in a control INS-1 cell. Scale bar = 10 μm. **D)** Example traces showing decreases in plasma membrane GFP-C1-PLCdelta-PH fluorescence intensity in response to carbachol in living INS-1 and RyR2^KO^ cells. **E)** Quantification (AUC) of the decrease in plasma membrane GFP-C1-PLCdelta-PH fluorescence in control INS-1 cells and RyR2^KO^ cells in the presence or absence of 100 μM 2-APB. Carbachol stimulated a greater decrease in PH-PLCδ-GFP fluorescence intensity in control INS-1 cells than in RyR2^KO^ cells (***, *P* < 0.001). 2-APB significantly inhibited this decrease in control INS-1 cells (****, *P* < 0.0001), but not in RyR2^KO^ cells. INS-1 cells + Carb: n = 27; RyR2^KO^ cells + Carb: n = 25; INS-1 cells + Carb + 2-APB: n = 18; RyR2^KO^ + Carb + 2-APB: n = 13. One-way ANOVA with Tukey’s multiples comparisons test.

Since muscarinic receptor-stimulated PLC activity appeared to be reduced in RyR2^KO^ cells, but not IRBIT^KO^ cells, as assessed with the IP_1_ assay, we examined the PIP_2_ levels in control INS-1, RyR2^KO^, and IRBIT^KO^ cells. Using immunofluorescence (primary antibody against PIP_2_) of fixed cells, we found that cellular PIP_2_ levels were significantly elevated in both IRBIT^KO^ and RYR2^KO^ cells compared to control INS-1 cells (Fig 3A). Therefore, lack of substrate (i.e. PIP_2_) doesn’t appear to account for the decreased muscarinic receptor-stimulated PLC activity in RyR2^KO^ cells. To complement the results of the IP_1_ assay, we measured PLC activity in live cells using a probe consisting of the Pleckstrin homology domain of PLC delta fused to GFP (GFP-C1-PLCdelta-PH) [33]. This probe binds to PIP_2_, and can be imaged selectively in the plasma membrane using total internal reflection fluorescence microscopy (TIRFm). Acute stimulation of PLC activity using 100 μM carbachol resulted in a rapid decrease in GFP fluorescence intensity detected by TIRFm in INS-1 cells expressing GFP-C1-PLCdelta-PH, reflective of the acute decrease in PIP_2_ concentrations in the plasma membrane as it is converted to diacylglycerol and IP_3_ (Fig 3D). Pre-treatment of INS-1 cells expressing GFP-C1-PLCdelta-PH with 100 μM 2-APB strongly inhibited the decrease in plasma membrane GFP fluorescence intensity stimulated by carbachol (Fig 3E). In contrast, carbachol stimulated a significantly smaller decrease in plasma membrane GFP fluorescence intensity in RyR2^KO^ cells expressing GFP-C1-PLCdelta-PH compared to that detected in control INS-1 cells (Fig 3E). Further, the decrease in GFP-C1-PLCdelta-PH plasma membrane fluorescence intensity stimulated by carbachol in RyR2^KO^ cells was not significantly inhibited by 100 μM 2-APB (Fig 3E). Thus, cellular PIP_2_ levels are increased in RyR2^KO^ and IRBIT^KO^ cells compared to control INS-1 cells, but PLC activity stimulated by carbachol is reduced in RyR2^KO^ cells compared to control INS-1 cells, and is insensitive to 2-APB.

### SOCE is impaired by deletion of RyR2, but not IRBIT

**Fig 4:**
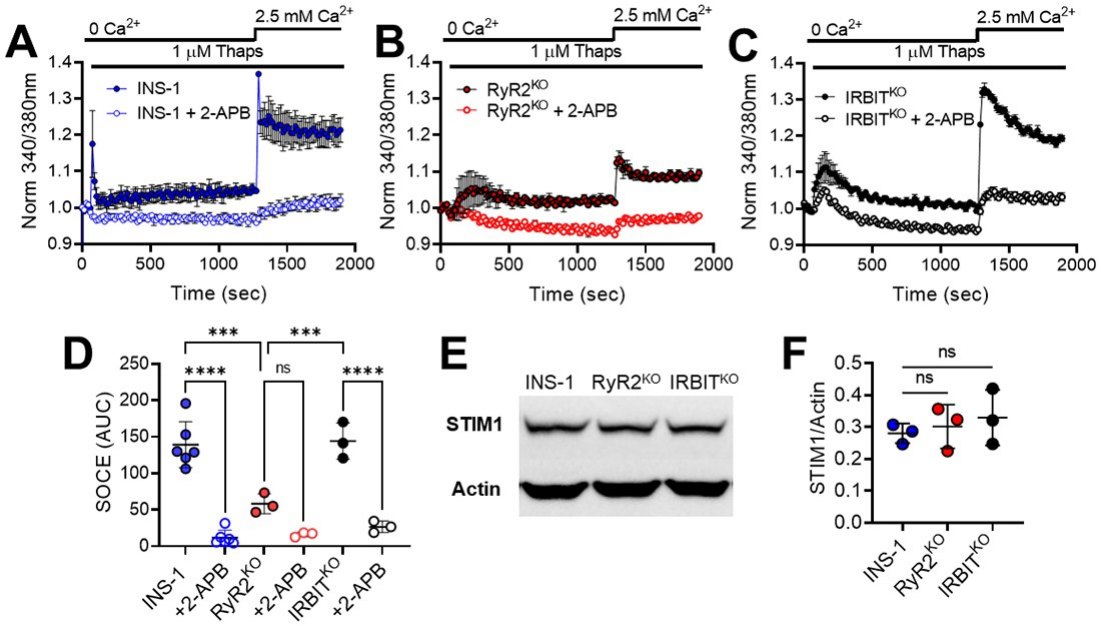
SOCE is diminished in the absence of RYR2, but not IRBIT. Representative experiments showing activation of SOCE in control INS-1 cells **A)**, in RyR2^KO^ cells **B)**, and in IRBIT^KO^ cells **C)**. ER Ca^2+^ stores were depleted with thapsigargin in the absence of extracellular Ca^2+^, and SOCE initiated by increasing extracellular Ca^2+^ to 2.5 mM. Each point is the mean of three replicates and is shown ± SE. **D)** Quantification of SOCE (AUC). The SOCE Ca^2+^ integral in significantly reduced in RyR2^KO^ cells compared to either control INS-1 cells or IRBIT^KO^ cells (***, *P* < 0.001). 2-APB (100 μM) significantly reduced SOCE in control INS-1 cells and IRBIT^KO^ cells, but not in RyR2^KO^ cells (****, *P* < 0.0001). Two-way ANOVA with Tukey’s multiple comparisons test. INS-1 cell: n = 6 separate experiments; INS-1 cells + 2-APB: n = 6 separate experiments; RyR2^KO^ cells: n = 3 separate experiments; RyR2^KO^ cells + 2-APB: n = 3 separate experiments. **E)** Immunoblot for STIM1 from cell lysates prepared from control INS-1, RyR2^KO^, and IRBIT^KO^ cells. Representative of three separate experiments. **F)** Quantification of STIM1 immunoblots from INS-1, RyR2^KO^, and IRBIT^KO^ cell lysates. The intensity of STIM1 bands was normalized to that of actin in each replicate (n = 3 for all cell lines). No significant difference in the STIM1/actin ratios among the three cell lines was detected (One-way ANOVA).

SOCE is an important mechanism that cells utilize to replenish internal stores of calcium, but is also implicated in maintenance of PLC activity during stimulation [29]. Therefore, we examined SOCE in RyR2^KO^, IRBIT^KO^, and control INS-1 cells to determine if a deficit in SOCE might explain the sharply decreased PLC activity in RyR2^KO^ cells. In this assay, fura2-AM was used to measure changes in [Ca^2+^]_in_ as cells were depleted of the ER Ca^2+^ by injection of 1 μM thapsigargin in the absence of extracellular calcium to activate the SOCE pathway, and twenty minutes later, 2.5 mM extracellular Ca^2+^ re-introduced to initiate Ca^2+^ influx via SOCE (Fig 1A). The magnitude of the response was quantified by integrating the [Ca^2+^]_in_ over time for the 10 minutes after addition of 2.5 mM Ca^2+^ (Fig 4D). In INS-1 cells, this protocol produced a robust SOCE response that was strongly inhibited by pretreatment of cells with 100 μM 2-APB (Fig 1D). In contrast, the magnitude of the SOCE response detected with this protocol was significantly reduced in RyR2^KO^ cells, and was insensitive to 2-APB (Fig 4D). However, the magnitude of the SOCE response in IRBIT^KO^ cells was not different from control INS-1 cells, and was strongly inhibited 2-APB (Fig 4D). STIM1 is an essential component of SOCE, and decreases in STIM1 lead to impairments in SOCE [23, 24]. Impairments in SOCE and reduced expression of STIM1 have been associated with β-cell dysfunction [24]. Therefore, we performed semi-quantitative immunoblotting to assess protein levels of STIM1 in RyR2^KO^, IRBIT^KO^, and control INS-1 cells, and found no difference between the three cell lines (Fig 4F). 2-APB is reported to have multiple targets [41] and to induce mitochondrial swelling [42], so we performed the SOCE assays under conditions that minimized exposure of the cells to 2-APB. In this modified assay, 2-APB was co-applied to cells with thapsigargin, and extracellular Ca^2+^ was added back to cells 5 minutes after thapsigargin + 2-APB application. The resulting [Ca^2+^]_in_ responses in the presence or absence of 2-APB were quantified as before. This modified assay essentially reproduced the results in Fig 4 (i.e. RyR2^KO^ cells have impaired SOCE compared to INS-1 cells and IRBIT^KO^ cells) except that SOCE in RyR2^KO^ cells was inhibited by 2-APB (S1 Fig). Thus, deletion of RyR2 leads to reduced SOCE in INS-1 cells, while deletion of IRBIT is without effect on SOCE. This deficit in SOCE in RyR2^KO^ cells is likely not due to a reduction of STIM1 protein levels.

### Voltage-gated calcium channel current density is elevated in RyR2^KO^cells

**Fig 5:**
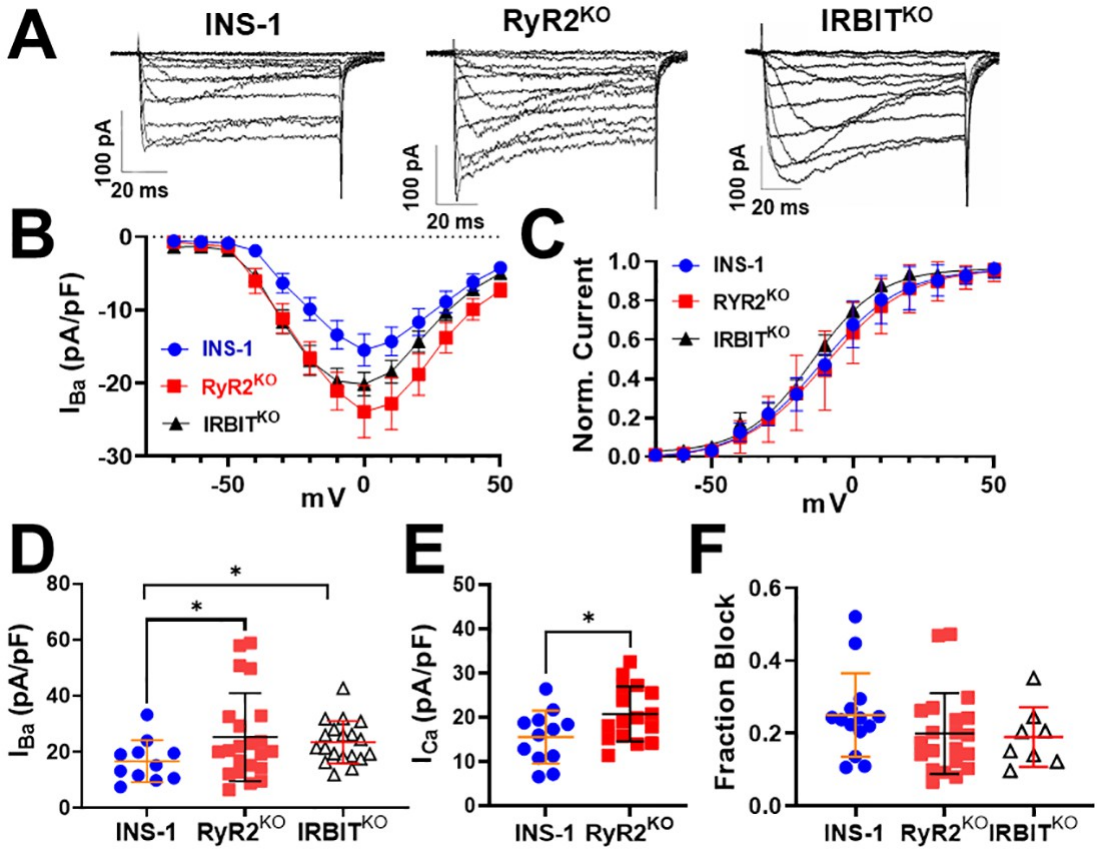
Deletion of RyR2 or IRBIT increases Ca_v_ current density. **A)** Example barium current ensembles, recorded from the indicated cell types, elicited by stepping to voltages ranging from -70 mV to +50 mV for 100 ms from a holding potential of -80 mV. **B)** Barium current-voltage relationship of complied data from the indicated cell types. Data are shown as mean ± SE. **C)** Voltage-dependence of activation (V_1/2_ act.) was not different between INS-1 (-9.3 ± 1.2 mV; n = 11) and RyR2^KO^ (-7.5 ± 1.0 mV; n = 24), or IRBIT^KO^ cells (-13.7 ± 1.2 mV; n = 19) (One-way ANOVA). **D)** Peak barium current (I_Ba_) density of RyR2^KO^ and IRBIT^KO^ cells was greater than that of control INS-1 cells (*, *P* < 0.05; Welch’s unpaired t-test). INS-1 cells: n = 11; RyR2^KO^ cells: n = 22; IRBIT^KO^ cells: n = 19. **E)** Peak calcium current (I_Ca_) density was RyR2^KO^ cells was great than that of control INS-1 cells (*P* < 0.05; Welch’s unpaired t-test). INS-1 cells: n = 12; RyR2^KO^ cells: n = 16. **F)** The fraction of current blocked by 5 μM nifedipine wasn’t different between INS-1 and RyR2^KO^ cells, or between INS-1 and IRBIT^KO^ cells (Welch’s unpaired t-test). INS-1 cells: n = 14; RyR2^KO^ cells: n = 21; IRBIT^KO^ cells: n = 8.

PIP_2_ is known to be an essential regulator of channel function at the plasma membrane, including regulation of voltage-gated calcium channels [43, 44]. Given that RyR2^KO^ cells have reduced basal and stimulated PLC activity, and IRBIT^KO^ and RyR2^KO^ cells have increased PIP_2_ levels, we examined the Ca_v_ current density in RyR2^KO^, IRBIT^KO^, and control INS-1 cells using whole-cell voltage clamp. Current traces were recorded from all three cell lines using Ba^2+^ as the permeant ion (Fig 5A). Plots of the barium current density (pA/pF) voltage relationship for each cell type are summarized in Fig 5B. We examined the voltage-dependence of activation of Ca_v_ current from all three cell lines, and found no difference between either RyR2^KO^ cells or IRBIT^KO^ cells and controls cells (Fig 5C). Peak barium current density was greater for both RyR2^KO^ and IRBIT^KO^ cells compared to control INS-1 cells (Fig 5D). Further, the increase in peak current density in RyR2^KO^ cells versus control cells was also observed when Ca^2+^, rather than Ba^2+^, was used as the permeant cation (Fig 5E). To determine the percentage of current contributed by L-type channels (Ca_v_1.2 and Ca_v_1.3), we measured the fraction of current blocked by a maximally effective concentration (5 μM) of the L-type channel selective inhibitor, nifedipine. The fraction of current blocked by nifedipine in control INS-1 cells was ~24%, and was not significantly different in RyR2^KO^ or IRBIT^KO^ cells (Fig 5F). These results suggest a general upregulation of Ca_v_ channel activity in RyR2^KO^ and IRBIT^KO^ cells, since the fraction of current contributed by L-type channels and non-L-type channels remains unchanged.

### Hydrolysis of PIP_2_ preferentially reduced current in RyR2^KO^ cells

**Fig 6:**
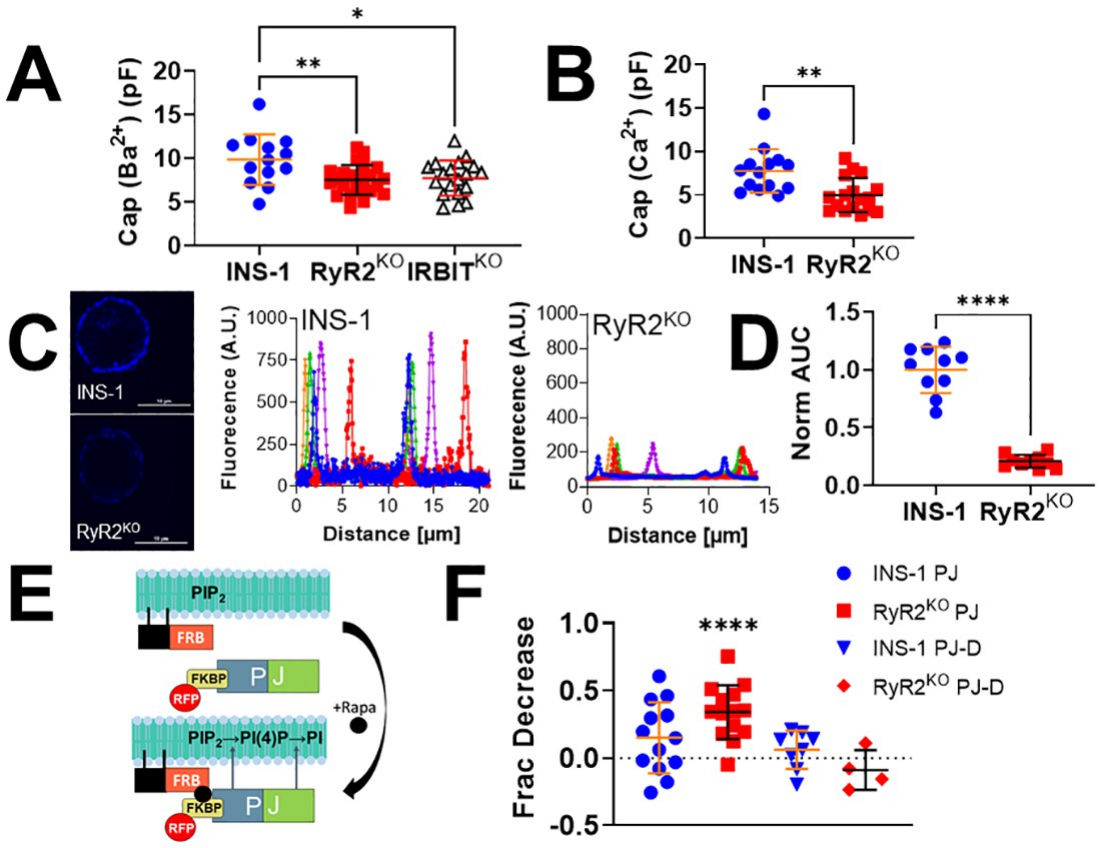
Increased plasma membrane PIP_2_ levels may contribute to the increased Ca_v_ channel activity in RyR2^KO^ cells. **A)** Whole-cell membrane capacitance of INS-1, RyR2^KO^, and IRBIT^KO^ cells measured in extracellular solution containing 10 mM BaCl_2_. (*, *P* < 0.05, **, *P* < 0.01, One-way ANOVA with Dunnette’s post-hoc test). INS-1: n = 13 cells; RyR2^KO^: n = 24 cells; IRBIT^KO^: n = 19 cells. **B)** Whole-cell membrane capacitance of INS-1 and RyR2^KO^ cells measured in extracellular solution with 10 mM CaCl_2_. (**, *P* < 0.01, Welch’s unpaired t-test). INS-1: n = 14 cells; RyR2^KO^: n = 17 cells. **C)** Cortical f-actin levels are reduced in RyR2^KO^ cells compared to controls. Fluorescence intensity of phallodin-CF405 was detected by confocal microscopy, and quantified using line scans of stained cells. Scale bars = 10 μm. **D)** Comparison of phalloidin-CF405 fluorescence intensity in RyR2^KO^ cells and INS-1 cells, normalized to INS-1 cells. Cortical CF405 fluorescence was significantly lower in RyR2^KO^ cells compared to control INS-1 cells (****, *P* < 0.0001; un-paired t-test). INS-1 cells: n = 10; RyR2^KO^ cells: n = 10. **E)** Diagram of the pseudojanin (PJ) lipid phosphatase system [34]. Rapamycin induces dimerization of FK506 binding protein 12 (FKBP), and fragment of mTOR that binds rapamycin (FRB). FRB is fused to the plasma membrane-localizing peptide lyn_11_, and dimerization drives FKBP-PJ fusion to the plasma membrane. PJ thus localized, rapidly degrades PIP_2_ in the plasma membranes. Cells expressing these constructs can be identified by detection of red fluorescent protein (RFP) emission. **F)** Rapamycin perfusion (1 μM) reduces current compared to baseline in RyR2^KO^ cells expressing PJ but not in INS-1 cells expressing PJ, or in either cell line expressing the phosphatase-dead PJ construct PJ-D. (****, *P* < 0.0001; one sample t-test). INS-1 cells + PJ: n = 13; INS-1 cells +PJ-D: n = 8; RyR2^KO^ cells + PJ: n = 14; RyR2^KO^ cells + PJ-D: n = 4.

Given the increased Ca_v_ current density measured in both RyR2^KO^ and IRBIT^KO^ cells, we sought to identify a likely mechanism to explain it. Membrane capacitance is a proxy for cell surface area, and is used to normalize current amplitude to cell size. Whole-cell membrane capacitance of RyR2^KO^ and IRBIT^KO^ cells, measured in extracellular solution containing 10 mM BaCl_2_, and RyR2^KO^ cells measured in 10 mM CaCl_2_, was significantly reduced compared to control INS-1 cells (Fig 6A & 6B). Thus, RyR2^KO^ and IRBIT^KO^ cells maintain similar levels of whole-cell current as control INS-1 cells despite reduced cell surface area. PIP_2_ is a key regulator of actin polymerization [45], and cortical actin filaments (f-actin) are known to positively regulate voltage-gated calcium channel activity [46]. Therefore, control INS-1 and RyR2^KO^ cells were fixed, stained with phalloidin-CF405, and imaged with confocal microscopy to measure relative amounts of cortical f-actin (Fig 6C). Surprisingly, RyR2^KO^ cells had ~80% lower intensity of cortical f-actin staining compared to control INS-1 cells (Fig 6D). To further assess the contribution of PIP_2_ to the increase in Ca_v_ current density, RyR2^KO^ and control cells were transfected with 2 plasmids encoding a rapamycin-inducible phosphatase system (Fig 6E). In the presence of rapamycin, the lipid phosphatase pseudojanin (PJ), fused to FKBP, localizes to the plasma via a membrane-anchored co-receptor (FRB) for rapamycin, and rapidly depletes PIP_2_ [34]. Application of 1 μM rapamycin to cells expressing these plasmids reduced current amplitude from baseline in RyR2^KO^ cells, but not in control INS-1 cells (Fig 6F). Application of rapamycin to cells expressing pseudojanin lacking phosphatase activity (PJ-D) failed to reduce current amplitude from baseline in either control or RyR2^KO^ cells (Fig 6F). Thus, deletion of RyR2 results in greatly reduced levels of cortical f-actin which may contribute to the decreased membrane capacitance in RyR2^KO^ cells compared to control INS-1 cells. However, hydrolysis of PIP_2_ rapidly reduces Ca_v_ current in RyR2^KO^ cells but not in control INS-1 cells, suggesting that PIP_2_ potentiates Ca_v_ channel activity, and therefore Ca_v_ current density, in RyR2^KO^ cells.

### Deletion of RYR2 increases action potential frequency

**Fig 7:**
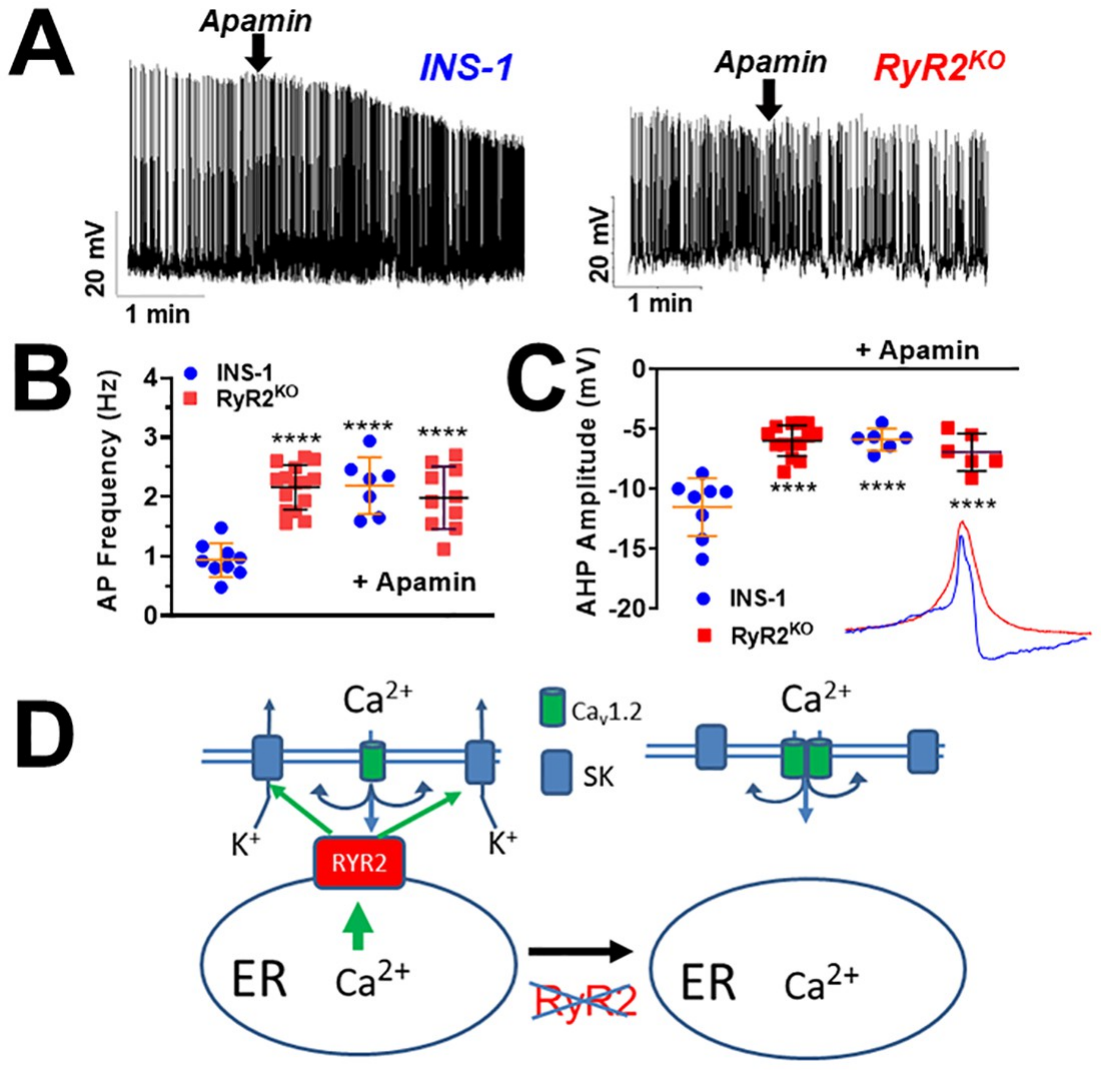
Deletion of RyR2 inhibits activation of SK channels during glucose stimulated electrical activity. **A)** Example trains of glucose-stimulated (18 mM) action potentials in INS-1 and RyR2^KO^ cells. The SK channels blocker apamin (1 μM) was added at the time indicated by the arrows. **B)** Glucose-stimulated action potentials were more frequent in RyR2^KO^ cells than in control INS-1 cells. Addition of apamin increased action potential frequency 2-fold in control INS-1 cells, but had no effect on action potential frequency in RyR2^KO^ cells. (****, P < 0.0001; One-way ANOVA with Tukey’s multiple comparisons test) INS-1 control: n = 9 cells; RyR2^KO^ control: n = 15 cells; INS-1 + apamin: n = 7 cells; RyR2^KO^ + apamin: n = 10 cells **C)** Afterhyperpolarization (AHP) amplitude of glucose-stimulated action potentials is reduced in RyR2^KO^ cells compared to control INS-1 cells. Apamin reduces AHP amplitude in control INS-1 cells, but not in RyR2^KO^ cells. (****, *P* < 0.0001; One-way ANOVA with Tukey’s multiple comparisons test). Inset- overlay of action potentials from a control INS-1 cells (blue) and an RyR2^KO^ cells (red) illustrates the difference AHP amplitude. INS-1 control: 9310 action potentials from 8 cells; RyR2^KO^ control: 21,241 action potentials from 14 cells; INS-1 + apamin: 5371 action potentials from 6 cells; RyR2^KO^ + apamin: 7018 action potentials from 6 cells. **D)** Model for RyR2 control of glucose-stimulated action potential frequency. In control cells, Ca^2+^ release from RyR2 induced by Ca^2+^ influx from voltage-gated Ca^2+^ channels actives SK channels during glucose stimulation. In the absence of RyR2, the enhanced Ca^2+^ influx (i.e. increased Ca_v_ channel current density and increased action potential frequency) isn’t able to activate SK channels. However, the reduced SOCE observed in RyR2^KO^ cell could also account for the deficit in SK channel activation in these cells.

Given the changes in Ca_v_ channel current density observed in RyR2^KO^ cells, we examined the excitability of both control INS-1 cells and RyR2^KO^ cells by measuring changes in membrane potential in response to elevated glucose concentrations using current clamp in the zero-current injection (I = 0) mode. To elicit depolarization and firing of action potentials (AP), cells under current clamp were perfused with a solution containing 2.5 mM glucose to establish a baseline membrane potential, then switch to a solution containing 18 mM glucose (Fig 7A). Average action potential frequencies were determined using uniform bursts of action potentials and excluding gaps between bursts. We found that the glucose-stimulated action potential frequency is doubled by RyR2 deletion. Control INS-1 cells displayed a mean action potential frequency of 0.94 Hz while that in RyR2^KO^ cell was 2.16 Hz (Fig 7B). Moreover, when 1 μM apamin, a blocker of the SK (KCa2) channel) [47]., was applied to control INS-1 cells during a train of action potentials, the firing rate increased to 2.18 Hz (Fig 7B). In contrast, application of 1 μM apamin to RyR2^KO^ cells during a train of action potentials had no effect on firing frequency (Fig 7B). The main mechanism whereby apamin enhances firing frequency in β-cells is by reducing the SK channel-mediated afterhyperpolarization (AHP) at the end of each action potential [5]. Therefore, we measured the AHP amplitudes in both control INS-1 and RyR2^KO^ cells by measuring the difference between the lowest potential and the previous plateau potential. The AHP amplitudes measured for INS-1 and RyR2^KO^ cells were: -11.52 mV and -5.98 mV, respectively in the absence of 1μM apamin, -5.38 mV and -6.96 mV, respectively, in the presence of 1μM apamin (Fig 7C). Thus, the increased glucose-stimulated action potential frequency and reduced AHP amplitude in RyR2^KO^ cells, along with their unresponsiveness to apamin, argue that SK channels aren’t activated during glucose stimulation in RyR2^KO^ cells (Fig 7D).

## Discussion

### Role of RyR2 in pancreatic β-cells

The role of RyRs in pancreatic β-cells has been the subject of much debate. Evidence for CICR stimulated by Ca^2+^ influx via Ca_v_ channels was reported over twenty years ago [3], and various roles for RyRs in β-cells have been reported [9, 48, 49]. Genetic studies in humans revealed that patients with mutations in RyR2 associated with severe arrhythmias are also prone to glucose intolerance [50]. In addition, knock-in of these gain of function mutations in mice also results in glucose intolerance [50]. Recently, RyR2 protein was detected in both INS-1 cells and murine β-cells using the highly sensitive and specific targeted mass spectrometry technique [10], and RyR2 was determined to be the major RyR transcript present in highly purified human β-cells [8]. Peptides derived from the intracellular II-III loop of Ca_v_1.2 modulate RyR2 activity [51], and expression of a peptide corresponding to the entire II-III loop of Ca_v_1.2 disrupts CICR in INS-1 cells [5]. Previously, we found that deletion of RyR2 from INS-1 cells abolishes the Ca^2+^ transient stimulated by caffeine, suggesting that RyR2 is the major functional RyR in these cells [11]. RyR2 deletion from INS-1 cells also strongly reduced insulin content, transcript, and basal and glucose stimulated secretion, and markedly reduced levels of the protein IRBIT [11]. In this study, we examined the role of RyR2 and IRBIT in regulation of key Ca^2+^ signaling processes in INS-1 cells.

### RyR2 regulates tolbutamide-stimulated Ca^2+^ transients and insulin secretion

Depolarization-dependent Ca^2+^ influx is a key driver of insulin secretion [52]. Tolbutamide stimulates a biphasic Ca^2+^ response, with a rapid peak, followed by sustained plateau of elevated [Ca^2+^]_in_ (Fig 1A). This rapid peak was inhibited by ryanodine (Fig 1A) [53] and by a peptide derived from an intracellular loop of Ca_v_1.2 [5]. RyR2 deletion similarly abolished the rapid peak in [Ca^2+^]_in_ in response to stimulation by tolbutamide as assessed by a plate-based assay with a short time window (2 min) (Fig 1B). However, in single-cell experiments with a longer time frame (5 min), tolbutamide stimulated marked peaks in [Ca^2+^]_in_ in both RyR2^KO^ and IRBIT^KO^ cells that were delayed (by 110 sec and 53 sec, respectively) compared to the rapid peak observed in control INS-1 cells. This delay corresponds with the contribution of IP_3_R to the Ca^2+^ transient in RyR2^KO^ and IRBIT^KO^ cells but not in control INS-1 cells (Fig 1E). Even though the Ca^2+^ integral in response to tolbutamide wasn’t reduced by RyR2 deletion, basal and tolbutamide-stimulated insulin secretion were reduced 53 and 64%, respectively, compared to control INS-1 cells (Fig 1F). This deficit in secretion observed in RyR2^KO^ cells persisted upon co-stimulation with an EPAC-selective cAMP analog and tolbutamide (42% reduction compared to control INS-1 cells). These deficits in tolbutamide-stimulated insulin secretion mirror those reported for glucose-stimulated insulin secretion in RyR2^KO^ cells [11]. Since tolbutamide directly inhibits the K_ATP_ channel [54], it is unlikely that deficits in glucose-stimulated insulin secretion in RyR2^KO^ cells reflect a deficit in glucose metabolism, but likely result from the marked reduction in insulin transcript and content in these cells [11]. This conclusion is also supported by the observation that stimulation of electrical activity by glucose in RyR2^KO^ cells is intact, with a higher firing rate than control INS-1 cells (Fig 7). Similarly, the reduction in tolbutamide-stimulated insulin secretion in IRBIT^KO^ cells mirrors the deficit in glucose-stimulated insulin secretion reported for these cells [11]. Given that the Ca^2+^ integral upon tolbutamide stimulation in IRBIT^KO^ cells isn’t different (Fig 1E), and the Ca_v_ channel current density was increased compared to control INS-cells (Fig 6), it is also likely that the deficits in insulin secretion stimulated by tolbutamide (Fig 1F) or glucose in IRBIT^KO^ cells results from decreased insulin transcript and content [11]. However, even though an EPAC-selective cAMP analog significantly potentiated tolbutamide-stimulated insulin secretion in all three cells lines (Fig 1F), and activation of EPAC2 strongly potentiates stimulated insulin granule transport to the plasma membrane and fusion [55], a deficit in granule trafficking upon RyR2 or IRBIT deletion can’t be ruled out.

### RyR2 regulates SOCE and PLC activity

The deletion of RyR2 and IRBIT both permitted activation of IP_3_R receptors during tolbutamide stimulation (Fig 1E), and increased the effectiveness of 50 μM bethanechol in stimulating increases in [Ca^2+^]_in_ compared to control INS-1 cells (Fig 2B). Given this, we examined the activation of PLC by the muscarinic receptor agonist carbachol. A positive feedback mechanism whereby Ca^2+^ release via IP_3_R directly, or indirectly via depletion of ER Ca^2+^ and activation of SOCE, is proposed to support sustained PLC activity [29]. Our finding that PLC activation in response to glucose is reduced in both RyR2^KO^ and IRBIT^KO^ cells, with a significantly greater deficit in RyR2^KO^ cells (Fig 2C), suggests that under these conditions, the reduced PLC activation mirrors deficits in secretion. It is possible insulin [56] or ATP [57] secretion by β-cells has an autocrine effect via activation of PLC. It will be of interest to determine if either of these mechanisms can account for this observation. In contrast, the stimulation of PLC by carbachol was sharply reduced in RyR2^KO^ cells, but not different from controls in IRBIT^KO^ cells as assessed with the IP_1_ accumulation assay (Fig 2C). The marked effect of RyR2 deletion on carbachol-stimulated PLC activity was further corroborated using TIRFm imaging of GFP-C1-PLCdelta-PH at the plasma membrane (Fig 3C–3E). In both assays, the SOCE inhibitor 2-APB strongly inhibited PLC activation in control INS-1 cells. Therefore, we examined SOCE stimulated by the sarco/endoplasmic reticulum Ca^2+^-ATPase inhibitor thapsigargin. The marked inhibition of SOCE specifically in RyR2^KO^ cells suggests that SOCE plays a role in maintaining PLC activity in response to muscarinic receptor activation, in agreement with Thore et al. [29]. Moreover, these results suggest that RyR2 plays a key role in maintaining SOCE, as proposed by Lin et al. [27], via a mechanism that doesn’t involve regulation of STIM1 protein levels (Fig 4E and 4F). However, it is not clear from our studies how or if RyR2 deletion interferes with functional coupling of STIM1 to SOCE. The decrease in SOCE activity could potentially account for the sharply reduced basal insulin secretion in RyR2^KO^ cells, since SOCE is increased during ER stress leading to enhanced basal insulin secretion [58]. Our finding that deletion of RyR2 reduces the magnitude of SOCE in INS-1 cells is consistent with Gustafsson et al. [59], who found that RyR activation stimulates Ca^2+^ influx via 2-APB-sensitive TRP channels in INS-1E cells. Experiments utilizing 2-APB must be interpreted cautiously, since it can inhibit IP_3_ receptors and Ca^2+^-ATPases, in addition to SOCE channels, and can induce mitochondrial swelling [42]. However, in this study, the effects of RyR2 deletion on the magnitude of the SOCE and PLC activity were evident in the absence of 2-APB, and it was used in both control and RyR2^KO^ cells to inhibit SOCE and PLC activity in well-established assays.

### RyR2 regulates PIP_2_ levels and Ca_v_ channel current

Influx of Ca^2+^ via voltage-gated Ca^2+^ channels is a key regulator of insulin secretion since inhibitors of both L-type [4, 60] and non-L-type [4] channels are able to substantially inhibit secretion. Our finding that Ca_v_ channel current density is upregulated in RyR2^KO^ and IRBIT^KO^ cells (Fig 5D & 5E) contrasts with our finding that insulin secretion in these cell lines is reduced (Fig 1F). The lack of any significant change in activation potential (Fig 5C), or in the fraction of current blocked by the L-type channel inhibitor nifedipine (Fig 5F), suggest a general increase in Ca_v_ channel activity. However, it is possible that the complement of L-type (i.e. Ca_v_1.2, Ca_v_1.3) or non-L-type channel subtypes (Ca_v_2.2, Ca_v_2.3) has changed without changing the ratio of nifedipine-sensitive to nifedipine-insensitive current. A prominent role of plasma membrane PIP_2_ levels in maintenance of Ca_v_ current is supported by the experiments with the rapamycin-activated phospholipid phosphatase pseudojanin [34], showing a preferential reduction of I_Ba_ upon depletion of PIP_2_ in RyR2^KO^ cells over control INS-1 cells. We propose that the increase in Ca_v_ channel current density results, in part, from increased PIP_2_ levels at the plasma membrane (Fig 3B). PIP_2_ is a potentiator of Ca_v_ channels activity in general [61], and specifically in pancreatic β-cells [31]. In the case of RyR2^KO^ cells, the increased PIP_2_ levels could follow from sharply reduced PLC activity (Figs 2B and 3E). We speculate that the decrease in surface area and cortical f-actin observed in RyR2^KO^ cells could result from decreased exocytosis, as reflected by the marked decrease in basal and stimulated insulin secretion observed in these cells (Fig 1F). Exocytosis provides phospholipids for expansion of the plasma membrane, via fusion of vesicles, and promotes f-actin assembly at the membrane [62]. One limitation of this study is that changes in Ca_v_β auxiliary subunit protein levels haven’t been ruled out as a mechanism contributing to the increase in Ca_v_ current density, since this mechanism could also potentially increase plasma membrane surface expression of Ca_v_ channels, regardless of subtype. However, the mechanism by which Ca_v_β subunits promote retention of Ca_v_ channels at the plasma membrane is proposed to require binding to f-actin [46]. Given the marked reduction in cortical f-actin in RyR2^KO^ cells, it seems unlikely that increased levels of Ca_v_β subunits play a key role in maintaining Ca_v_ channels at the plasma membrane in the face of reduced whole-cell capacitance (i.e. surface area) in these cells.

A limitation of this study is the low-resolution method used to measure PIP_2_ levels (i.e. immunocytochemistry). Our finding that PLC activity is sharply reduced and Ca_v_ current is strongly potentiated by PIP_2_ in RyR2^KO^ cells supports the data suggesting that PIP_2_ levels are increased in these cells. Deletion of IRBIT apparently increases cellular PIP_2_ levels as well, and this could be ascribed to the decreased basal and glucose-stimulated PLC activity observed in this cell line (Fig 2B). However, IRBIT also binds to the catalytic core of phosphatidylinositol phosphate kinases (PIPK) type Iα and type IIα [63], key enzymes in the production of PIP_2_. This binding is competitive with Mg^2+^, ATP, and phosphatidylinositol-4-phosphate, but had no discernable effect on kinase activity *in vitro*. We speculate that, in cells, IRBIT binding may negatively regulate PIPK such that deletion or reduction of IRBIT enhances plasma membrane PIP_2_ levels. It will be of interest to use other, more direct approaches to assess the effect of RyR2 or IRBIT deletion specifically on plasma membrane PIP_2_ levels in β-cells.

### RyR2 regulates glucose-stimulated electrical activity

Glucose-stimulated action potentials in pancreatic β-cells are mediated by the membrane depolarizing effect of K_ATP_ channel closure upon ATP binding. As the membrane potential is depolarized, Ca_v_ channels are activated, leading to the upstroke of the action potential, followed by activation of K_v_ channels which repolarize the membrane potential [64]. This process is fine-tuned by small conductance, Ca^2+^-activated K^+^ (SK) channels (K_Ca_2.1–3) [47]. Activation of SK channels regulates action potential frequency by conducting a Ca^2+^-dependent activation of K^+^ efflux late in the repolarization phase, referred to as the afterhyperpolarization (AHP) [65]. The magnitude of the AHP controls AP frequency because it largely dictates the time required for spontaneous depolarization to reach the threshold for firing. We previously demonstrated that overexpression of the Cav1.2 II-III intracellular loop in INS-1 cells increases action potential frequency through disruption of CICR and SK channel activity [5]. In this study, we found that deletion of RyR2 from INS-1 cells has the same effect on glucose-stimulated action potential frequency. In RyR2^KO^ cells, glucose stimulated action potentials with a mean frequency of 2.2 Hz, and these action potentials were insensitive to the SK channel blocker apamin (Fig 7B). In contrast, the mean frequency in control INS-1 cells was 0.94 Hz, which was increased to 2.3 Hz in the presence of apamin. Similarly, the AHP amplitude in RyR2^KO^ cells was -6.0 mV in the absence of apamin, and -7.0 mV in the presence of apamin. In contrast, the AHP amplitude in the absence of apamin in control INS-1 cells was -11.5 mV, and -5.9 mV in the presence of apamin (Fig 7C). Thus, both the frequency of action potentials and the amplitude of the AHP in RyR2^KO^ cells in the absence of apamin were indistinguishable from those in the presence of apamin in both RyR2^KO^ and control cells, indicating a failure in SK channel activation in RyR2^KO^ cells. As expected, apamin significantly increased action potential frequency and decreased AHP amplitude in control INS-1 cells. These results suggest that RyR2 plays an important role in activating SK channels in INS-1 cells (Fig 7D). A limitation of this study is that the mechanism by which RyR2 activates SK channels remains unclear. It is unlikely that Ca^2+^ influx via Ca_v_ channels plays a role since Ca_v_ current density is increased in RyR2^KO^ cells. It is also unlikely that the Ca^2+^ content of the ER plays a role, since it was not different between control and RyR2^KO^ cells [11]. We speculate that local Ca^2+^ signaling in the proximity of SK channels may be mediated by Ca^2+^ release via RyR2 or by Ca^2+^ influx via SOCE. Finally, the possibility that one or more of the three SK channel subtypes present in INS-1 cells [66] is downregulated in RyR2^KO^ cells, can’t be ruled out.

## Conclusions

**Fig 8:**
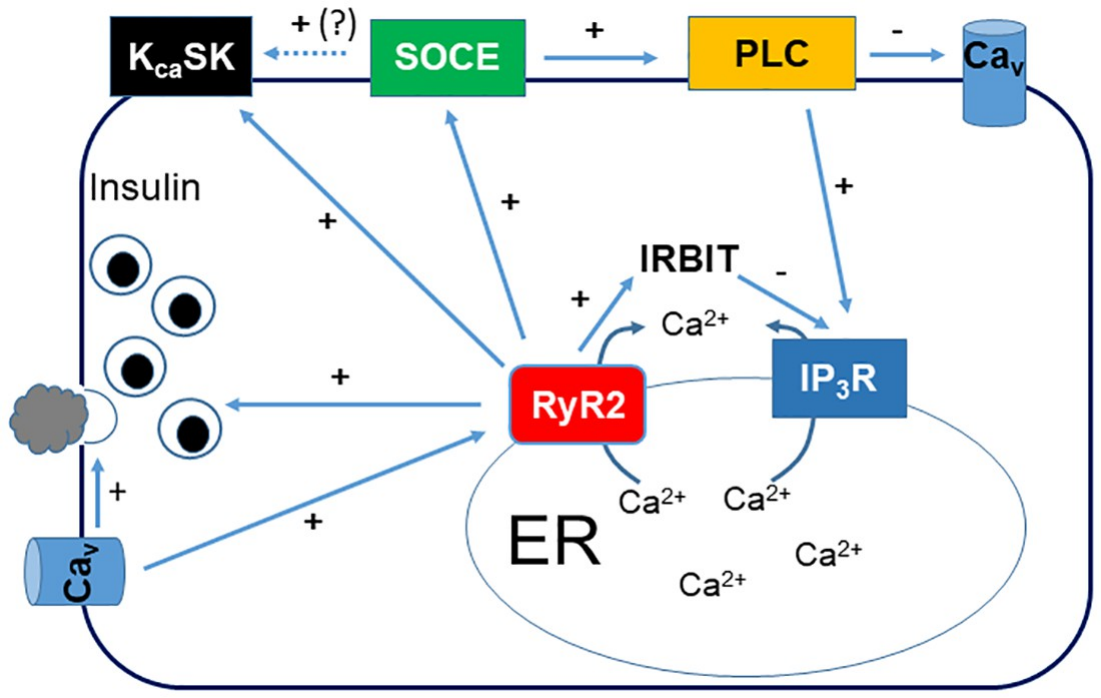
Model for regulation of Ca^2+^ dynamics in pancreatic β-cells. RyR2 is regulated by influx of Ca2+ via L-type Ca_v_ channels [3, 5], which can also directly stimulate rapid insulin release [67]. RyR2 regulates insulin secretion either directly via Ca^2+^ release from the ER or indirectly via regulation of insulin content and transcript level [11]. RyR2 plays a key role in regulating SOCE via a yet unknown mechanism. Maintenance of SOCE is critical for basal and stimulated PLC activity. Reduced PLC activity in the absence of RyR2 increases plasma membrane PIP_2_ levels, and increases Ca_v_ channel activity. RyR2 also directly or indirectly (via regulation of SOCE) regulates activation of SK channels. Finally, RyR2 regulates IP_3_R-mediated Ca^2+^ release, and perhaps many other cellular processes, via regulation of IRBIT levels [11].

This study examined the role of RyR2 in several aspects of Ca^2+^ signaling in the pancreatic β-cell line INS-1. The major findings (Summarized in Fig 8) are that 1: Deletion of RyR2 delays the major peak in [Ca^2+^]_in_ in response to tolbutamide stimulation, and enhances release of Ca^2+^ through IP_3_ receptors; 2: Deletion of RyR2 markedly reduces basal insulin secretion and insulin secretion in response to tolbutamide stimulation; 3: Deletion of RyR2 markedly reduces basal and stimulated PLC activity; 4: Deletion of RyR2 reduces SOCE in response to emptying of ER Ca^2+^ stores with thapsigargin; 5: Deletion of RyR2 decreases whole-cell capacitance, but increases Ca_v_ channel current density potentially via an increase in plasma membrane PIP_2_ levels; 6: Deletion of RyR2 inhibits activation of SK channels and increases glucose-stimulated action potential frequency. It will be of interest to determine the molecular mechanisms by which RyR2 regulates these various aspects of Ca^2+^ signaling.

## Supporting information

S1 Fig Deletion of RyR2 reduces SOCE: Reduced time of exposure to 2-APB. Representative experiments showing activation of SOCE in control INS-1 cells **A)**, in RyR2^KO^ cells **B)**, and in IRBIT^KO^ cells **C)** with reduced time of exposure to 2-APB prior to re-introduction of Ca^2+^. ER Ca^2+^ stores were depleted with injection thapsigargin in the absence of extracellular Ca^2+^, and SOCE initiated by increasing extracellular Ca^2+^ to 2.5 mM. 100 μM 2-APB was co-injected with thapsigargin for some experiments prior to re-addition of Ca^2+^ to minimize off-target effects. Each point is the mean of three replicates and is shown ± SE. **D)** Quantification of SOCE (AUC). The SOCE Ca^2+^ integral in significantly reduced in RyR2^KO^ cells compared to either control INS-1 cells (***, *P* < 0.001). or IRBIT^KO^ cells (*, *P* < 0.05). Acute application of 2-APB (100 μM) significantly reduced SOCE in all cells (####, *P* < 0.0001). Two-way ANOVA with Tukey’s multiple comparisons test. Each bar represents the mean (± SD) of four separate experiments done in triplicate.
(TIF)

S1 Raw images (PDF)
