# Supplementary figures and images for: RyR2 regulates store-operated Ca2+ entry, phospholipase C activity, and electrical excitability in the insulinoma cell line INS-1
Source: PLoS One. 2023 May 4;18(5):e0285316. doi: 10.1371/journal.pone.0285316 (PMC10159205; doi:10.1371/journal.pone.0285316)

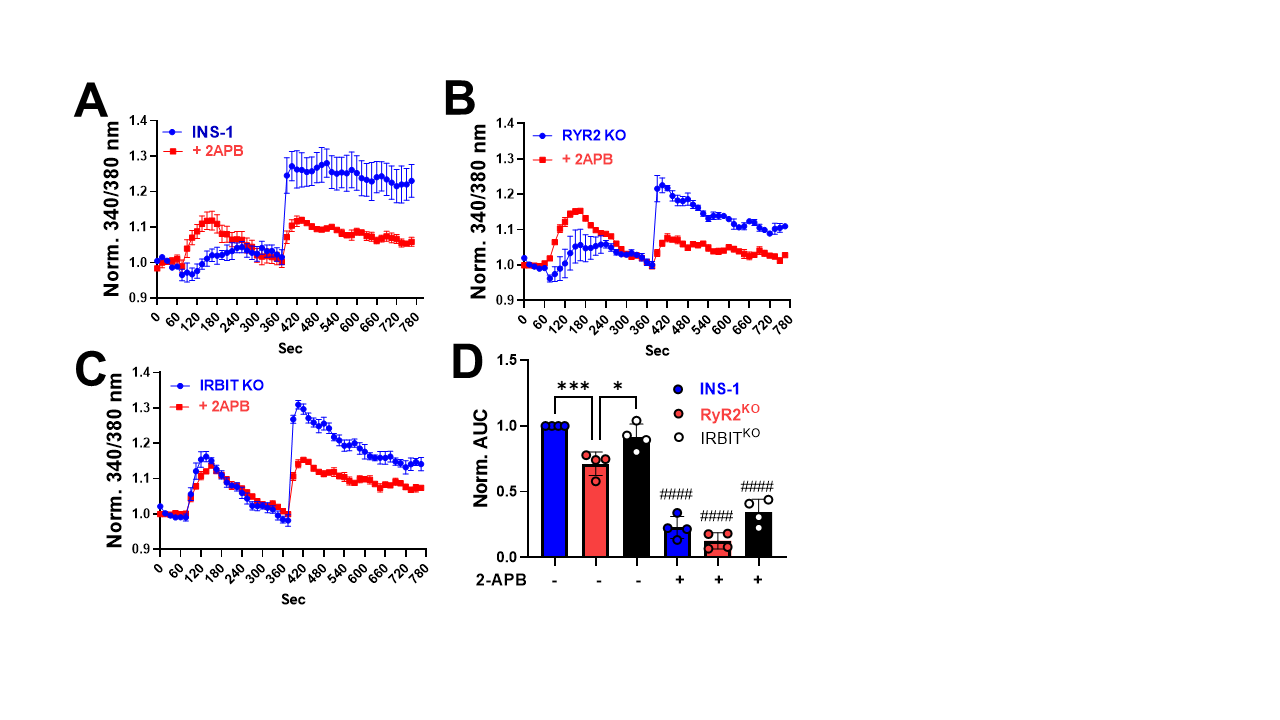

Supplement: S1 Fig — Representative experiments showing activation of SOCE in control INS-1 cells A), in RyR2KO cells B), and in IRBITKO cells C) with reduced time of exposure to 2-APB prior to re-introduction of Ca2+. ER Ca2+ stores were depleted with injection thapsigargin in the absence of extracellular Ca2+, and SOCE initiated by increasing extracellular Ca2+ to 2.5 mM. 100 μM 2-APB was co-injected with thapsigargin for some experiments prior to re-addition of Ca2+ to minimize off-target effects. Each point is the mean of three replicates and is shown ± SE. D) Quantification of SOCE (AUC). The SOCE Ca2+ integral in significantly reduced in RyR2KO cells compared to either control INS-1 cells (***, P < 0.001). or IRBITKO cells (*, P < 0.05). Acute application of 2-APB (100 μM) significantly reduced SOCE in all cells (####, P < 0.0001). Two-way ANOVA with Tukey’s multiple comparisons test. Each bar represents the mean (± SD) of four separate experiments done in triplicate. (TIF) [file pone.0285316.s001.tif]
