# Supplementary material for: RyR2 regulates store-operated Ca2+ entry, phospholipase C activity, and electrical excitability in the insulinoma cell line INS-1
Source: PLoS One. 2023 May 4;18(5):e0285316. doi: 10.1371/journal.pone.0285316 (PMC10159205; doi:10.1371/journal.pone.0285316)
Supplement: S1 Raw images — (PDF) [file pone.0285316.s002.pdf]

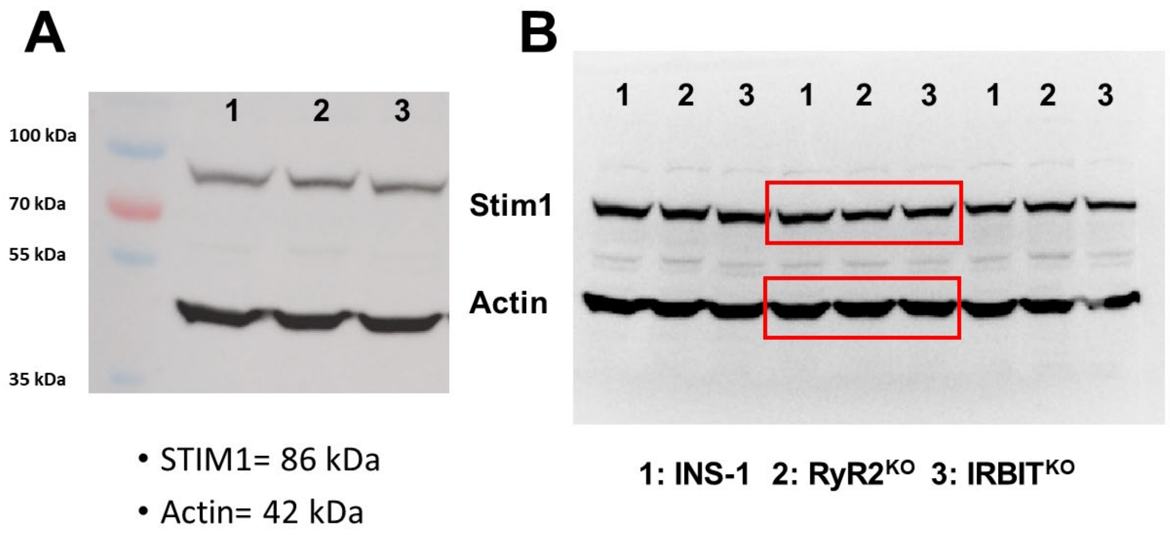

Full-length immunoblots of actin and stim1 in control INS-1, RyR2^{KO}, and IRBIT^{KO} cells with molecular weight markers. A) Overlay image of membrane showing pre-stained molecular weight markers and protein bands detected by chemiluminescence after immunoblotting for actin and stim1. The image isolates the molecular weight standards and the left-most set of sample lanes for clarity. Size of molecular weight markers is indicated (kDa). **B)** Image of bands detected by chemiluminescence for the entire blot shown partially in **A**. All three sets of bands for actin and stim1 were used for quantitation in Fig 4F. The bands shown in Fig 4E are indicated by red rectangles.
